# Effectiveness of intra-venous steroids for preventing surgery for lumbo-sacral radiculopathy secondary to intervertebral disc herniation: a retrospective study of 213 patients

**DOI:** 10.1038/s41598-022-10659-1

**Published:** 2022-04-23

**Authors:** Daniel Kovarsky, Adi Shani, Alon Rod, Dan Ciubotaru, Nimrod Rahamimov

**Affiliations:** 1 Bar-Ilan University Medical School, Tzfat, Israel; 2 Department of Orthopedics B and Spine Surgery, Galilee Medical Center, Nahariya, Israel

**Keywords:** Health care, Medical research, Neurology

## Abstract

The natural history of lumbar disc herniation with radiculopathy is favorable, with 95% of patients expected to be pain-free within 6 months of onset. Despite the favorable prognosis, operative treatment is often chosen by patients unable to “ride out” the radicular episode. Prospective studies comparing surgical with non-surgical treatment have demonstrated similar long-term results. We conducted a retrospective case-series study of patients with a lumbar disc herniation and intractable radicular pain without significant neurological deficits treated with intra-venous dexamethasone. The primary outcome measure was whether the patient had undergone operative treatment within 1 year of receiving the intravenous steroid treatment. 213 patients met our inclusion criteria. 30 were lost to follow-up and 2 had died before completing 1 year of follow-up. Of the remaining 181 patients, 133 (73.48%) had not undergone surgery within 1 year of receiving intra-venous steroid treatment while 48 (26.51%) had undergone surgery. 6 (3.31%) of the patients had undergone surgery more than 1 year of receiving IV steroid treatment. Intravenous steroid treatment in our retrospective series was approximately 30% better at preventing the need for surgery than the reported outcomes of conservative treatment in randomized controlled trials previously published.

## Introduction

It has been known for many years that acute radicular pain due to lumbar disc herniation carries a favorable prognosis, regardless of treatment^1–4^. Hakelius in 1970, in a retrospective comparison of 583 patients undergoing conservative or surgical treatment, found that 96% of the patients treated surgically and 89% of the patients treated conservatively, were free of symptoms within 6 months of onset^1^. Gugliotta et al., in a prospective trial of 370 patients, all potential candidates for surgical treatment and allocated to surgical or conservative treatment by their treating physicians, found significantly better reduction in radicular pain at 3 weeks in the surgically treated group, but in the 3 month to 2 year follow-up there was no difference between the groups^4^. Weinstein et al. in the Spinal Patient Outcomes Research Trials (SPORT), found in 501 patients randomly allocated to either conservative or surgical treatment in 13 participating centers, no statistically significant difference in all primary outcome measures between the groups for up to 2 years of follow-up^5^. The study had a high 1-year crossover rate, with 41% of patients allocated to the surgical treatment group undergoing conservative therapy, and 43% of patients allocated to the conservative treatment group undergoing surgery. Similar findings and crossover rate were also noted by Peul et al.^6^.

The term ‘disc herniation’ along with the term ‘sciatica’ are used colloquially to signify leg pain originating from a lumbar spine pathology. While in essence this is correct, many spinal and extra-spinal pathologies can produce similar symptoms, therefore spine professionals need better terminology enabling diagnosis of a much more specific pathology, for effective treatment to be offered^7^. Hence, in addition to the spine level, disc pathology is classified as bulge or herniation or extrusion or sequestration, the location as central, lateral, foraminal or extra-foraminal (“far lateral”) and the sciatica as L3 or L4 or L5 or S1 radiculopathy^8^. Only when all of these parameters correlate, an invasive treatment such as lumbar discectomy can be successful^7^. An erroneous or non-specific diagnosis may lead almost invariably to sub-optimal surgical outcomes.

While surgical treatment for lumbar disc herniation is limited to a few surgical options, the term ‘conservative therapy’ encompasses a vast array of options, none of them shown to be significantly more effective than another, or provide better short-term outcomes superior to the improvement expected with the passage of time from onset^3,9–11^. For the researcher seeking to study conservative treatment outcomes, this creates a methodological problem as there is no set benchmark for conservative treatment success to compare with. An alternative option is to evaluate the number of patients failing conservative treatment, a benchmark clearly set by the crossover rates in previously published randomized controlled trials^2,5,6,10,12^, thus making the comparison to the expected failure, clearly defined by opting for surgery.

One of the conservative treatment options is administration of glucocorticoid steroids. These can be delivered either orally, parenterally, intravenously or injected within the spinal structures^3,11,13^. Goldberg et al. reported moderate improvement in function and no difference in pain in a randomized prospective trial of 269 patients receiving either oral prednisone or placebo^13^. Lee et al., in a meta-analysis of 14 high quality randomized controlled trials (RCT’s) pooling 1502 patients receiving either epidural steroids or placebo/local anesthetic/saline, found a statistically significant reduction in pain, but not in function, at 1 month and 3 months, with a non-significant difference at 6 months for the steroid group^14^. Aminmansour et al. reported a significant reduction in postoperative radicular pain in patients receiving intravenous 40/ 80 mg dexamethasone intraoperatively vs. placebo in an RCT of 61 patients^15^. Finckh et al. in a study of 65 patients randomized to receive either IV 500 mg methylprednisolone or placebo found a significant difference of small magnitude in the reduction of leg pain in the steroid group during the first 3 days, but this effect did not persist^16^.

The optimal end result of conservative therapy is complete resolution of symptoms without the need for surgery in a time-frame acceptable to the patient. Unfortunately, there is no universal agreement as to what this time-frame is, with recommendations for surgery ranging from 6 weeks to 12 months from symptom onset^11,17^.

Our spine unit is located in a 722 bed regional referral hospital, serving a population of approximately 650,000. It is common practice for family physicians and community orthopedic surgeons to refer to our emergency room patients with intractable radiculopathy due to an established lumbar disc herniation that have failed conservative ambulatory treatment. Most are rapidly discharged after receiving analgesic therapy, and patients with severe or progressive neurological symptoms are operated on an emergent basis, but patients without significant neurological deficits, unable to be discharged from the emergency room, are hospitalized for IV steroid therapy as part of our usual treatment protocol.

Having intractable pain and failing ambulatory conservative treatment essentially means that all of this patient subset consists of potential surgical candidates, meeting similar inclusion criteriae to the patients randomized in previous studies comparing conservative to surgical treatment^2,4,5,10,18^.

The aim of this study was to determine the percentage of patients opting for surgical treatment within 1 year of receiving IV steroids for lumbar radiculopathy secondary to a disc herniation and compare the group that eventually underwent surgery with the group that did not.

## Materials and methods

This was a retrospective cohort study utilizing data retrieved from our hospital’s electronic database and a telephone survey.

To achieve a power of 80% (Based on 2-tailed Independent sample t-test, Alpha = 5%, effect size 0.2), a minimum of 160 patients were needed, calculated using the Gpower software^19^.

Included were all patients aged 18 and over, admitted to our spine surgery unit between August 2014 and January 2018 for treatment of intractable lumbar radicular pain clinically corresponding to a disc herniation or extrusion demonstrated on recent imaging (level, location and side-matched pain).

Excluded were patients discharged without completing the IV steroid course for any reason other than having surgery or patients that were retrospectively found to have had another underlying concurrent pathology producing the radicular clinical symptoms.

All patients received a single daily IV dexamethasone (Dexacort forte, Teva, Israel/Hungary) 1 mg/kg up to a daily maximum of 60 mg for six consecutive days. The dosage was tapered as follows: 100% on the first and second days, 50% on the third and fourth days, 25% on the fifth and sixth days, and then discontinued. IV proton pump inhibitors or H2 receptor antagonists were given throughout the duration of the steroid therapy, and additional analgesics (acetaminophen and/or dipyrone and/or tramadol or narcotics) provided if asked for pro re nata (p.r.n). Non-steroidal anti inflammatory drugs (NSAIDS) were not given due to the possibility of synergic erosive gastritis with concurrent high-dose steroids.

All patients included in the study were reviewed to determine if they had undergone surgery within 1 year of receiving IV steroid therapy. If their electronic records did not indicate they had been operated at our hospital, they were contacted by telephone and asked by if they had undergone surgery for their lumbar radiculopathy elsewhere.

Statistical analysis was performed using IBM SPSS Statistics software version 25. Quantitative data was presented as mean ± SD, median and range. Qualitative data was presented as frequencies and percentages. Quantitative data was compared between the groups using the Independent t-test or Mann–Whitney test, according to variable distribution. If the normality assumption was violated we chose the Mann–Whitney test. Qualitative data was compared with the Chi square test or Fisher's exact tests (if expectancy was < 5). All tests performed and all reported values were two-sided unless otherwise indicated. A p value less or equal to 0.05 was considered significant.

### Ethical approval

This study was conducted according to the principles of the declaration of Helsinki and approved by the Galilee Medical Center medical ethics review board (0010-18-NHR, Israel ministry of health registration 20182062).

### Consent to participate

A full waiver of informed consent was approved by the Galilee Medical Center medical ethics review board (0010-18-NHR, Israel ministry of health registration 20182062) Since it was done retrospectively on unidentified data obtained from patient medical records. All patients contacted by phone were informed as to the nature of the study and gave their consent to participate.

### Consent for publication

Only anonymous patient data is reported, therefore no consent for publication was required.

## Results

213 consecutive patients answering our inclusion criteria were found. 30 were not reviewed because they were lost to follow-up and/or did not have updated contact details. 2 patients, who had not undergone surgery, died within the 1 year post-treatment duration and were therefore excluded as well (see Table 1, detailed study flow diagram).

**Table 1:**
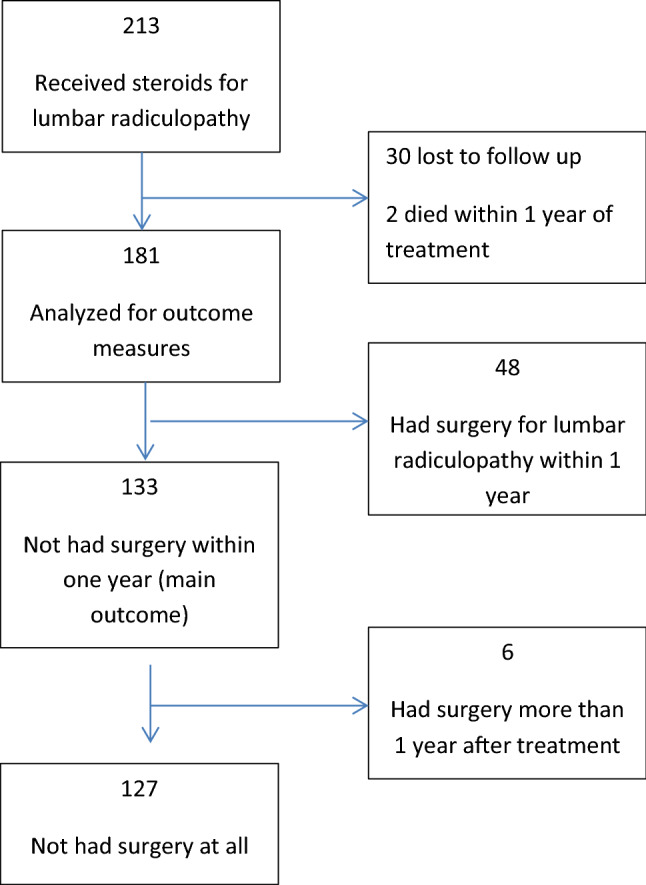
Flow diagram.

Of the remaining 181 patients, 133 (73.48%) had not undergone surgery within 1 year of receiving IV steroid treatment (the primary outcome measure), 48 (26.51%) had undergone surgery. 6 (3.31%) of the patients had undergone surgery more than 1 year of receiving IV steroid treatment.

No significant difference was found in the mean age, gender or comorbidities between the two groups (Tables 2,3).

**Table 2:** Demographics and total comorbidities of both groups.

	Not operated (N = 133)	Operated (N = 48)	P (2-sided)	Test
Age mean (SD)	56.65(13.638)	56.63(14.582)	0.990	Independent sample T-Test
**Gender**				
F N (%)	59 (44.4)	21 (43.8)	1.000	Chi-Square test
M N (%)	74 (55.6)	27 (56.3)		
**Total comorbidities**				
Mean	1.14	1.21	0.664	Mann Whitney Test
Median (min–max)	0 (0–9)	0.5 (0–5)

**Table 3:** Detailed comorbidities.

	Not operated within 1 year N(%)	Operated within 1 year N(%)	P (2-sided)	Test
Patients with one or more comorbidities	64 (48.1%)	24 (50.0%)	0.867	Chi-square test
Cardiac	12 (9.0%)	6 (12.5%)	0.574	Fisher's exact test
Vascular	5 (3.8%)	1 (2.1%)	1.000	Fisher's exact test
Hypertension	35 (26.3%)	15 (31.3%)	0.573	Chi-square test
Hyperlipidemia	28 (21.1%)	15 (31.3%)	0.169	Chi-square test
Diabetes mellitus	17 (12.8%)	8 (16.7%)	0.626	Chi-square test
Rheumatologic	4 (3.0%)	1 (2.1%)	1.000	Fisher's exact test
Malignancy	10 (7.5%)	0 (0.0%)	0.065	Fisher's exact test
Respiratory	7 (5.3%)	4 (8.3%)	0.485	Fisher's exact test
Hypothyroidism	6 (4.5%)	2 (4.2%)	1.000	Fisher's exact test
Urinary tract	2 (1.5%)	0 (0.0%)	1.000	Fisher's exact test
Osteoporosis	6 (4.5%)	0 (0.0%)	0.344	Fisher's exact test
Gastrointestinal	4 (3.0%)	2 (4.2%)	0.657	Fisher's exact test
Ophthalmologic	1 (0.8%)	1 (2.1%)	0.461	Fisher's exact test
Psychiatric	2 (1.5%)	1 (2.1%)	1.000	Fisher's exact test
Other	7 (5.3%)	1 (2.1%)	0.683	Fisher's exact test

No significant difference was found between the two groups.

## Discussion

The definition of treatment success or failure in a time-dependent self-resolving medical condition is difficult. As any treatment for radicular pain without neurological deficits must provide results that are better than the well-established excellent natural history^20^, the proposed therapy is expected to either shorten the clinical course and/or lower pain levels and/or produce better long term outcomes than other therapies.

Many outcome measures have been utilized to evaluate treatment success in previous studies, such as the length of the radicular episode, self-reported pain levels, return to work rate, physical and mental questionnaires, overall patient satisfaction and more^1,2,5,9–11,14,21^. While most of these measures are validated and comparable, it is difficult to set a clear value defining unequivocal success.

Another problem in evaluating the efficacy of radicular pain treatment is determining the denominator, since a substantial number of radicular episodes, probably most, will resolve before reaching the spine specialist who can suggest a surgical option^22^. It is therefore difficult to determine from what percentage of patients “over the radar” the treatment and control groups should be selected and success or failure decided.

To overcome this problem, we have defined all patients referred to our emergency department for treatment of intractable radicular pain as the denominator, seeing all of them as fulfilling the North American Spine Society (NASS) criteria for surgical treatment^11^, since they had already failed conservative therapy.

Determining the numerator is no less difficult as there is no agreed expected percentage of success or failure of conservative treatment. We have chosen the cross-over numbers reported in randomized controlled trials (RCT), as the numerator benchmark. Weinstein et al. in the SPORT trial^5^ found that 43% of the patients assigned to conservative care, crossed over and had surgery within 12 months, and that 59% of the patients randomized for surgical treatment underwent surgery by 12 months of randomization. Peul et al.^12^ in a similar study, found that 44% of patients randomized to conservative care had surgery eventually, the vast majority within 1 year. Since their selection criteria was generally similar to ours, all of our patients could be considered potential candidates for randomization in those trials, and the 43–44% cross-over rate could generate the expected numerator.

We have found that only 26.51% of our patient cohort had undergone surgery within 1 year and an additional 3.31% had surgery later than 1 year after IV steroid treatment. These numbers are approximately 30% lower than the expected 43–44%, making the 13% difference the possible treatment effect.

While we have no definite answer as to why IV steroid therapy in our series produced superior results when compared to other forms of conservative therapy, several speculations can be made.

Extruded intervertebral discs have been shown to leak proinflammatory cytokines TNF, IL‑1α, IL‑1β, IL‑6 and IL‑17 which contribute to the spinal nerve root irritation inducing radiculopathy^23^. As steroids have a potent anti-inflammatory effect, our 6 day long IV steroid protocol might induce a better local reduction of inflammation and/or secondary edema at the affected nerve root site than other forms of administration (IM/PO/epidural) which are given at lower doses and shorter durations.

Another possible explanation might be the simple passage of time. Acute radiculopathy is generally time-limited, regardless of treatment, but having intractable pain is a strong motivator for surgery in patients unable or unwilling to “ride it out”. If, instead of surgery, 6 days of hospitalization for analgesic treatment are offered, the radicular episode might be on the decline in some of the patients, enabling them to continue ambulatory conservative care.

Our study has several limitations. This is a single-center study and results might not be the same in other populations. Another limitation is possibly a selection bias, since having intractable acute pain can be associated with depression, anxiety and mood disorders causing poor coping rather than objective disability^24^. In this context, glucocorticoids might act by reducing anxiety through a central mechanism^25^. We have not utilized any of the personality questionnaires, and therefore cannot state whether this is the case or not. Another limitation is that this study was only aimed at evaluating the percentage of patients opting for surgery, and did not assess the functional outcome for comparison with other conservative treatment options or with surgical treatment. As this was a retrospective study of normal clinical practice in our department and not a prospective trial, only the steroid protocol was uniform and additional analgetic treatment was not uniform. This might lead to concerns that the additional analgesia and not the steroids was responsible for the effect. As current North American Spine Society guidelines have not found sufficient evidence in favor of any pharmacological intervention over another^11^ in the treatment of lumbar radiculopathy, we assume, with caution, these variations did not have any effect on the rate of surgery within 1 year—the main treatment outcome.

In conclusion, we have found that high-dose IV steroids are an effective treatment for acute radicular pain, reducing by 30% the number of patients undergoing surgery within 1 year when compared to data from previously published RCT’s. Further studies are needed to determine the mechanism of this effect and it’s generalizability.

## Data Availability

All data generated or analysed during this study are included in this published article.
